# CA916798 predicts poor prognosis and promotes Gefitinib resistance for lung adenocarcinoma

**DOI:** 10.1186/s12885-023-10735-3

**Published:** 2023-03-23

**Authors:** Jian He, Xi Lan, Xiayan Liu, Caixia Deng, Hu Luo, Yan Wang, Ping Kang, Zhijian Sun, Lintao Zhao, Xiangdong Zhou

**Affiliations:** 1 Department of Respiratory medicine, The First Hospital Affiliated to Army Medical University, 29 Gaotanyan Main Street, Chongqing, 400038 China; 2 Institute of Pathology and Southwest Cancer Center, Southwest Hospital, Third Military Medical University (Army Medical University), Chongqing, 400038 China; 3 K2 Oncology Co., Ltd, Beijing, 100176 China

**Keywords:** Lung adenocarcinoma, CA916798, CCNB1, G2/M phase, Gefitinib resistance

## Abstract

**Background:**

Our previous studies have identified CA916798 as a chemotherapy resistance-associated gene in lung cancer. However, the histopathological relevance and biological function of CA916798 in lung adenocarcinoma (LUAD) remains to be delineated. In this study, we further investigated and explored the clinical and biological significance of CA916798 in LUAD.

**Methods:**

The relationship between CA916798 and clinical features of LUAD was analyzed by tissue array and online database. CCK8 and flow cytometry were used to measure cell proliferation and cell cycle of LUAD after knockdown of CA916798 gene. qRT-PCR and western blotting were used to detect the changes of cell cycle-related genes after knockdown or overexpression of CA916798. The tumorigenesis of LUAD cells was evaluated with or without engineering manipulation of CA916798 gene expression. Response to Gefitinib was evaluated using LUAD cells with forced expression or knockdown of CA916798.

**Results:**

The analysis on LUAD samples showed that high expression of CA916798 was tightly correlated with pathological progression and poor prognosis of LUAD patients. A critical methylation site in promoter region of CA916798 gene was identified to be related with CA916798 gene expression. Forced expression of CA916798 relieved the inhibitory effects of WEE1 on CDK1 and facilitated cell cycle progression from G2 phase to M phase. However, knockdown of CA916798 enhanced WEE1 function and resulted in G2/M phase arrest. Consistently, chemical suppression of CDK1 dramatically inhibited G2/M phase transition in LUAD cells with high expression of CA916798. Finally, we found that CA916798 was highly expressed in Gefitinib-resistant LUAD cells. Exogenous expression of CA916798 was sufficient to endow Gefitinib resistance with tumor cells, but interference of CA916798 expression largely rescued response of tumor cells to Gefitinib.

**Conclusions:**

CA916798 played oncogenic roles and was correlated with the development of Gefitinib resistance in LUAD cells. Therefore, CA916798 could be considered as a promising prognostic marker and a therapeutic target for LUAD.

**Supplementary Information:**

The online version contains supplementary material available at 10.1186/s12885-023-10735-3.

## Background

Lung cancer is the most common malignant tumor and the leading cause of cancer-associated deaths worldwide [1, 2]. Small cell lung cancer and non-small cell lung cancer (NSCLC) are two histopathological types of lung cancer seen in patients clinically. NSCLC accounts for approximately 85% of lung cancer and can be further classified into lung adenocarcinoma (LUAD, 40–50%), lung squamous cell carcinoma (about 30%), and large-cell carcinoma according to histopathological features [3–5]. As the major histological subtypes of NSCLC, LUAD generally evolves from mucosal glands and related with chronic inflammation in lungs [6, 7]. LUAD is one of the most aggressive and rapidly fatal types of cancer with overall survival of less than 5 years for LUAD patients [8, 9]. Despite the progression of chemo-radio therapy, targeted therapy and immunotherapy, the prognosis of LUAD is still poor with 5-year survival less than 15% [10, 11]. The low survival rate is mainly attributed to the primary or acquired resistance to treatments, resulting in progression, metastasis, and recurrence of LUAD [12, 13].

CA916798 gene is firstly identified by our group in a cisplatin-resistant LUAD cell line through suppression cut hybridization technology [14]. CA916798 is expressed at higher levels in the 12-week fetal lung, the 12-week fetal liver and embryonic skin tissues than in normal adult lung tissues in our previous experiments. These results indicate that the CA916798 may be related to embryonic development, which is a characteristic of most tumor-related genes. Indeed, the gene was expressed in a variety of tumor tissues and tumor cells, including lung cancer cells [15–18], prostate cancer cells [19, 20], renal cell carcinoma [21], and breast cancer [22]. Multiple roles of this gene have been reported. For example, our previous work indicated that high CA916798 gene expression was related to cisplatin resistance in LUAD and small cell carcinoma [15, 23, 24]. Accumulating evidence suggests that CA916798 has oncogenic roles. CA916798 is found to be regulated by PI3K/AKT and SHP2 pathways [17, 25, 26], which intensively promote proliferation and inhibit apoptosis in cancers. CA916798 promotes growth and metastasis of androgen-dependent prostate cancer cells [19]. In addition, C19orf48 encodes a minor histocompatibility antigen in renal cell carcinoma cells [21]. However, the clinical significance and biological function of CA916798 in LUAD remains unclear.

EGFR strongly stimulates cell proliferation, invasion, and metastasis via AKT/MTOR and MAPK/ERK signaling axes [27]. Constitutive activation mutations of EGFR have been frequently detected in LUAD patients, especially in east Asian countries, and these patients are shown to benefit from the first-generation tyrosine kinase inhibitors (TKIs), such as gefitinib and erlotinib [28]. Gefitinib is the most efficient treatment for blocking EGFR activation in clinical cases [29]. However, gefitinib resistance is inevitably developed in most patients [30, 31]. Although different mechanisms of acquired EGFR-TKIs resistance have been reported [32, 33], it remains poorly understood.

In this study, we further explored the functions of CA916798 in LUAD, which demonstrated critical involvements of CA916798 in poor prognosis of LUAD and revealed CA916798 as a promising target for in Gefitinib-resistant LUAD.

## Methods

### Cell lines and reagents

A549, PC9 and HCC827 human LUAD cell lines (Shanghai Institute of Biochemistry and Cell Biology, Shanghai, China) were cultured in DMEM containing 10% fetal bovine serum (FBS) (16,140,071, ThermoFisher Scientific, Shanghai, China) at 37 °C and 5% CO_2_. Ro-33,066, Gefitinib, and sodium carboxymethyl cellulose (CMC-Na) was purchased from Selleck (Houston, TX, USA).

## Lentivirus, infection, and stable cell lines

Three short hairpin RNAs (shRNAs) targeting CA916798 and mock shRNA were inserted into lentivirus by GenePharma (Shanghai, China). The shRNA target sequences are listed in Table 1. Lentivirus containing human full-length CA916798 or empty vector as control were constructed by GenePharma (Shanghai, China). Lentivirus was used to infect cells following standard protocols and cells were selected using 5 µg/mL puromycin (Sigma, USA).

**Table 1:** Sequences of CA916798 shRNA control shRNA.

Plasmid	Sequence
LV3-Ctrl	TTCTCCGAACGTGTCACGT
LV3-CA916798-Homo-1	TGGAGGCCGTCTTGGAGAT
LV3-CA916798-Homo-2	GACCCAACCCAGTGCACAAGG
LV3-CA916798-Homo-3	TCCATACGCCACCGTGAGA

## Immunohistochemistry (IHC)

LUAD tissue microarray (HLugA180Su06) was purchased from Shanghai Outdo Biotech CO., LTD. (http://www.superchip.com.cn/index.html). IHC of tissue array and tumor xenografts was performed using the streptavidin-biotin peroxidase complex method and a rabbit polyclonal antibody against CA916798 (1:100, Invitrogen, USA). Photographs were obtained using an Olympus BX51 microscope and expression was scored in five random fields using the Image-Pro Plus 5.0 software. The area sum and integrated optical density (IOD) sum of the positive-stained sites (brown staining) were measured in pixels. CA916798 expression intensity was determined as the mean value of IOD sum/area sum in five images per slide. The same parameter settings were maintained for all images. Combined with the clinical prognosis of patients, the cut-off value for expression was 0.027609, as analyzed with SPSS 22.0. Expression > 0.027609 was defined as CA916798 high expression.

## RNA extraction and quantitative real-time RT-PCR

Total RNA was extracted using the RNAfast200 Kit (Feijie, Shanghai, China) and the PrimeScript RT Master Mix (Takara, Japan) was used for reverse transcription according to the manufacturers’ instructions. qRT-PCR reactions were conducted with the SYBR Premix Ex Taq II (Takara) on the Bio-Rad CFX96 Real-Time PCR Detection System (Bio-Rad, USA) in accordance with the manufacturers’ instructions. qRT-PCR was performed in triplicate. β-actin mRNA expression was used for normalization. The qRT-PCR primers are listed in Table 2.

**Table 2:** Primer sequences used in the experiments

Gene symbol	Primer sequence
CA916798	Forward: 5’-TTCTCCGAACGTGTCACGT-3’
CA916798	Reverse: 5’-TGGAGGCCGTCTTGGAGAT-3’
ACTB	Forward: 5’-CATGTACGTTGCTATCCAGGC-3’
ACTB	Reverse: 5’-CTCCTTAATGTCACGCACGAT-3’

## Western blot analysis

Cells were lysed in RIPA buffer (Thermo, USA) containing 1% protease and phosphatase inhibitors following the manufacturer’s protocol. Lysates were incubated with SDS-PAGE Sample Loading Buffer (Bio-Rad, USA) for 10 min at 95 °C. Proteins were separated by 10% SDS-PAGE and transferred to a PVDF membrane (Bio-Rad, USA). After blocking in 5% non-fat milk for 2 h, the membrane was incubated at 4 °C overnight with the following primary antibodies: CA916798 (1:100, Invitrogen, USA), β-actin (1:5000, CST, USA) ,WEE1 (1:500, CST, USA), CDK1 (1:1000, CST, USA), p-CDK1Y15 (1:500, CST, USA), and CCNB1 (1:500, CST, USA).

## Cell invasion and migration assays

Transwell inserts (8.0 μm pore; Millipore, USA) were precoated with Matrigel (for invasion assays) or left untreated (for migration assays) and placed into 24-well plates. Cells were seeded into the upper chambers (5 × 10^4^/well) with 200 µL serum-free medium and 600 µL DMEM medium containing 10% FBS was added to the lower chambers. Cells were incubated for 36 h and 24 h for invasion and migration assays, respectively. Cells were then fixed in 4% paraformaldehyde and dyed with crystal violet. Five random fields were examined for each chamber, and the invaded or migrated cells were counted. Images were obtained using a light microscope at 100-fold magnification.

## Colony formation assay

Cells were seeded into 6-well plates at a density of 200 cells per well. Plates were then incubated at 37℃ with 5% CO_2_ for 10 ~ 14 days until colonies were visible. Plates were washed with phosphate buffer saline (PBS) and 4% paraformaldehyde was used to fix cells for 15 min. Colonies were stained with crystal violet solution for 15 min, followed by a wash in PBS and air-drying, five random fields were selected for each well and colonies were counted. Images were obtained using a light microscope at 100-fold magnification.

## Cell viability and flow cytometric analysis

Cells were seeded in a 96-well plate (1000 cells per well). Cell viability was measured over 7 days using the CCK8 Kit (Beyotime, Shanghai, China), according to the manufacture’s instruction. The Cell Cycle and Apoptosis Analysis Kit (Beyotime, Shanghai, China) was used to determine cell cycle distribution following the manufacturer’s instructions.

## Animal experiments

Six-week-old female NOD/SCID mice were obtained from Charles River Labs (Beijing, China) and kept in specific pathogen-free conditions with light/dark cycles of 12 h, 60% humidity, 23 ± 3 ℃, and free access to water. The mice were randomly divided into two groups: shRNA-CA916798, shRNA-control (n = 5 per group). The mice were anesthetized with an intraperitoneal injection of pentobarbital (50 mg/kg). No signs of peritonitis, pain, or discomfort were observed after anesthesia. The indicated cells were subcutaneously injected into mice (1 × 10^6^ cells per mouse). Tumor size was measured at days 15, 20, 24 and 28. For treatment, the Gefitinib and vehicle was given through gavage administration Qod for total 14 days. The mice were euthanized by cervical dislocation at 28 days after implantation. Tumors were fixed in formaldehyde and subjected to paraffin embedding for hematoxylin and eosin and IHC staining. The animal experiments were approved by the Laboratory Animal Welfare and Ethics Committee of Army Medical University (AMUWEC20182181).

## Statistical analysis

All experiments were repeated at least three times and each treatment was set up in triplicate, unless specially indicated otherwise. Data are presented as mean ± SD. Statistical analyses were performed using SPSS version 24.0 and GraphPad Prism 5, String Version 11.0 by a two-tailed Student’s t-test. The cut-off value of CA916798 IHC staining scores was analyzed with SPSS. Chi-square analysis was used to evaluate the relationship between CA916798 high rate and LUAD clinicopathological features. *P* < 0.05 indicated statistical significance. High and low expression of genes were defined using median value. TCGA_LUAD mRNA expression and methylation450K datasets were downloaded from https://xenabrowser.net. Gene Set Enrichment Assay (GSEA) (*P* < 0.05, FDR < 0.25) [34] were used to analyze data. The statistical significance was determined by Student’s t test and P < 0.05 was considered statistically significant. Kaplan-Meier survival plot sand log-rank statistics were used to evaluate the survival of patients. Pearson rank correlation was used to analyze the relationship between different genes or proteins.

## Results

**CA916798 overexpression predicted poor prognosis for LUAD patients.**

**Fig. 1:**
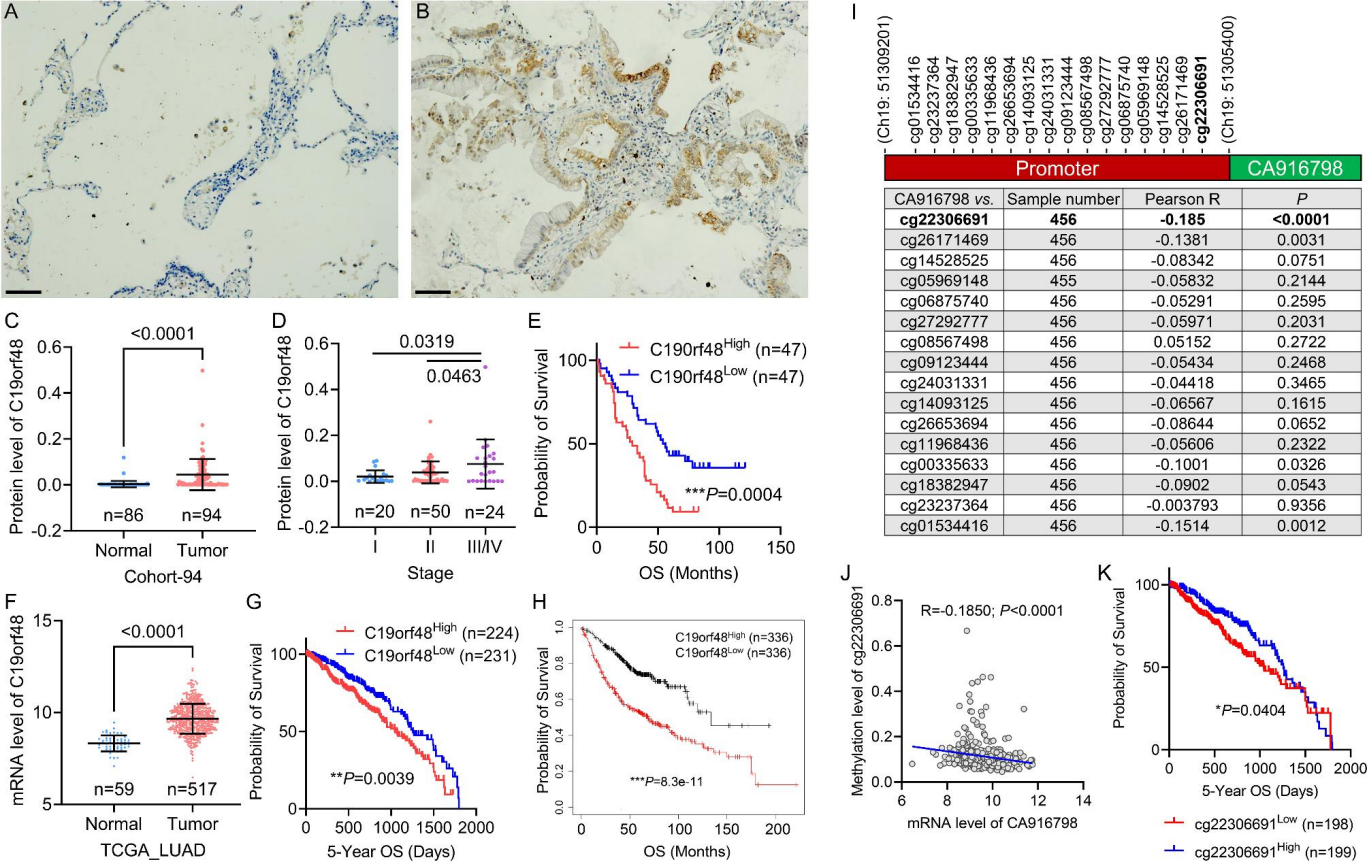
**CA916798 is upregulated in LUAD and predicts poor survival of patients** **A and B)** Representative IHC images of CA916798 in matched non-tumor tissue (A) and LUAD tissue (B). Scale bar = 100 μm. Brown color represents CA916798 protein; blue color represents nuclei **C)** IHC score of CA916798 expression in matched non-tumor tissue (Normal) and LUAD tissue (Tumor) from Cohort-94. Data are shown as mean ± SD. **D)** IHC score of CA916798 expression in different stages of LUAD from Cohort-94. Data are shown as mean ± SD. **E)** Kaplan-Meier analysis on overall survival (OS) in LUAD patients with CA916798^high^vs. CA916798^low^ from Cohort-94 **F)** mRNA level of CA916798 in matched non-tumor tissue (Normal) and LUAD tissue (Tumor) from TCGA_LUAD dataset. Data are shown as mean ± SD. **G)** Kaplan-Meier analysis on 5-year overall survival (OS) in LUAD patients with CA916798^high^vs. CA916798^low^ from TCGA_LUAD dataset **H)** Kaplan-Meier analysis on overall survival (OS) in LUAD patients with CA916798^high^vs. CA916798^low^ from KM-PLOT-LUAD database **I)** Pearson correlation list of methylation levels with CA916798 mRNA expression in TCGA_LUAD mRNA and methylation450K datasets **J)** Pearson correlation of CA916798 with cg22306691 from TCGA_LUAD mRNA and methylation450K datasets **K)** Kaplan-Meier analysis on 5-year overall survival (OS) in LUAD patients with cg22306691^high^vs. cg22306691^low^ from TCGA_LUAD methylation450K dataset

We first examined protein expression of CA916798 in 94 LUAD tumors and 86 matched non-tumor tissues by IHC. The result indicated that CA916798 was barely detected in non-tumor lung tissues (Fig. 1A) but strongly expressed in tumor cells of LUAD (Fig. 1B). Statistic data suggested that CA916798 expression was significantly higher in tumor tissues than in adjacent non-tumor tissues (Fig. 1C). The expression of CA916798 was correlated with tumor T-stage (Fig. 1D), but not with sex, age, N-stage, M-stage, and pathological grade **(data not shown)**. Moreover, patients with high CA916798 expression showed significantly shorter overall survival time than those with low CA916798 expression (Fig. 1E). To examine the results afore mentioned, we analyzed TCGA_LUAD dataset (gene expression RNAseq) and consistently observed the upregulation of CA916798 in tumor tissues compared with non-tumor lung tissues (Fig. 1F) and worse prognosis of patients with high CA916798 than those with low CA916798 (Fig. 1G). Analysis on Lung Cancer database from Kaplan-Meier plotter (https://kmplot.com) also proved that high expression of CA916798 was associated with shortened overall survival time of LUAD patients (Fig. 1H). Since CpG island methylation in gene promoter region has been known to be critically regulate gene expression, we analyzed the TCGA_LUAD methylation 450 K dataset. The result showed that several methylation probes were localized in the promoter of CA916798 gene and the one closely next to transcription start site (probe cg22306691) had significantly negative correlation with mRNA level of CA916798 (Fig. 1I J). In addition, the methylation level of cg22306691 was also dramatically downregulated in LUAD tissues compared to adjacent non-tumor tissues (Table 3). Moreover, low methylation level of probe cg22306691 predicted poor prognosis (Fig. 1K). Therefore, the expression of CA916798, partially regulated by promoter methylation, was correlated with tumor progression and predicted poor prognosis of LUAD.

**Table 3:** Average methylation level of LUAD vs. matched normal tissues in TCGA.

Probe	Average methylation level		LUAD:Normal	P
	Normal (n = 32)	LUAD (n = 456)		
**cg22306691**	**0.1336**	**0.1122**	**0.8400**	**0.0009**
cg26171469	0.1451	0.1425	0.9822	0.5224
cg14528525	0.0331	0.0315	0.9522	0.3985
cg05969148	0.1422	0.1275	0.8964	0.0003
cg06875740	0.1059	0.0898	0.8479	0.1407
cg27292777	0.0211	0.0209	0.9919	0.9088
cg08567498	0.0768	0.0750	0.9758	0.5768
cg09123444	0.0594	0.0568	0.9571	0.3367
cg24031331	0.0765	0.0800	1.0459	0.0915
cg14093125	0.0539	0.0508	0.9413	0.2544
cg26653694	0.0701	0.0666	0.9494	0.3165
cg11968436	0.0615	0.0579	0.9410	0.2271
cg00335633	0.0957	0.0947	0.9899	0.7703
cg18382947	0.1278	0.1253	0.9802	0.5676
cg23237364	0.0306	0.0332	1.0860	0.2523
cg01534416	0.2769	0.2327	0.8404	0.0001

**CA916798 promoted growth of LUAD cells*in vitro*and*in vivo***.

**Fig. 2:**
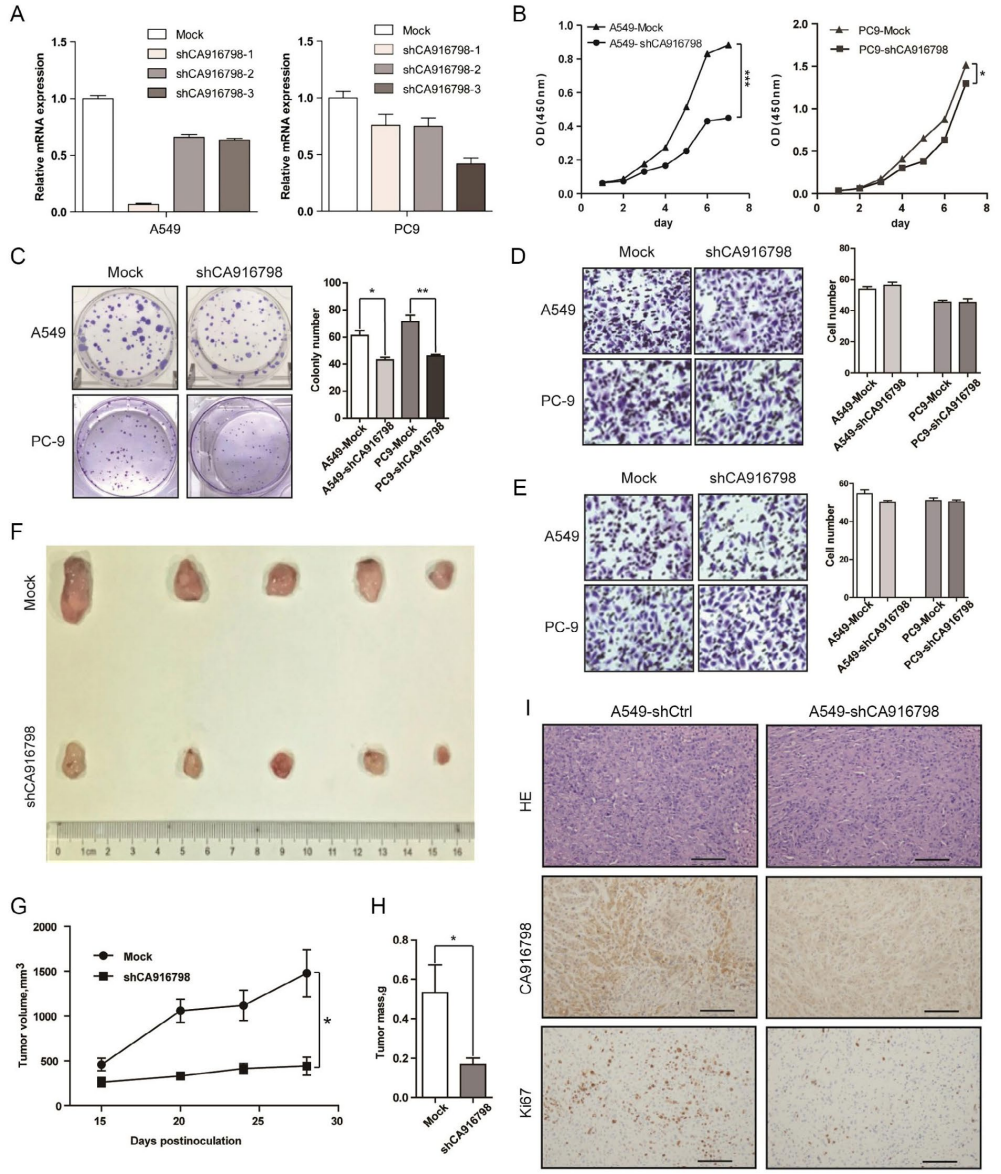
**CA916798 promotes growth of LUAD cells** ***in vitro*** **and** ***in vivo*** **A)** The efficiency of CA916798 knockdown in A549 and PC9 cells with three individual shRNA sequence targeting CA916798. shCA916798-1 and − 3 are used in A549 and PC9, respectively, for the following experiments **B)** Growth curve of CA916798-knockdown LUAD cells and mock cells as measured by CCK8 assay. Data are shown as mean ± SD (n = 3; *P < 0.05, ***P < 0.001) **C)** Colony formation of CA916798-knockdown LUAD cells and mock cells. Data are shown as mean ± SD (n = 3, *P < 0.05, ** *P* < 0.01) **D and E)** Measurement on migration (D) and invasion (E) in CA916798-knockdown cells and mock cells. Data are shown as mean ± SD (n = 3) **F-H)** Tumor image (F), tumor volume (G), and tumor weight (H) of xenograft models generated using A549-shCA916798 and mock cells. Data are shown as mean ± SD (n = 5, *P < 0.05) **I)** HE and IHC staining of tumor tissues from experimental mice obtained 28 days after implantation

We then explored the biological function of CA916798 in LUAD cells. For this purpose, CA916798 was stably knocked down in A549 and PC9 cell lines using shRNA targeting CA916798 (A549-shCA916798 and PC9-shCA916798) with scramble shRNA as control (A549-shCtrl and PC9-shCtrl) (Fig. 2A). CCK8 assay and colony formation assay indicated that CA916798 knockdown significantly impaired cell viability and growth (Fig. 2B C). However, transwell assay suggested that CA916798 knockdown did not affect cell migration (seeding cells without Matrigel) and invasion (seeding cells with Matrigel) of A549 and PC9 cells (Fig. 2D and E). To further examine the effects of CA916798 on cell growth of LUAD in vivo, we generated a subcutaneous xenograft model using A549-shCA916798 cells and A549-shCtrl cells. The result suggested that CA916798 knockdown suppressed tumorigenicity of A549 cells in nude mice (Fig. 2F H). Hematoxylin-eosin (HE) staining and Ki67 staining confirmed that CA916798 deficiency markedly reduced growth of A549 cells in vivo(Fig. 2I). Together, these results suggested that interference of CA916798 expression impaired cell growth of LUAD in vitro and in vivo.

**CA916798 was involved in regulation on G2/M phase transition in LUAD cells.**

**Fig. 3:**
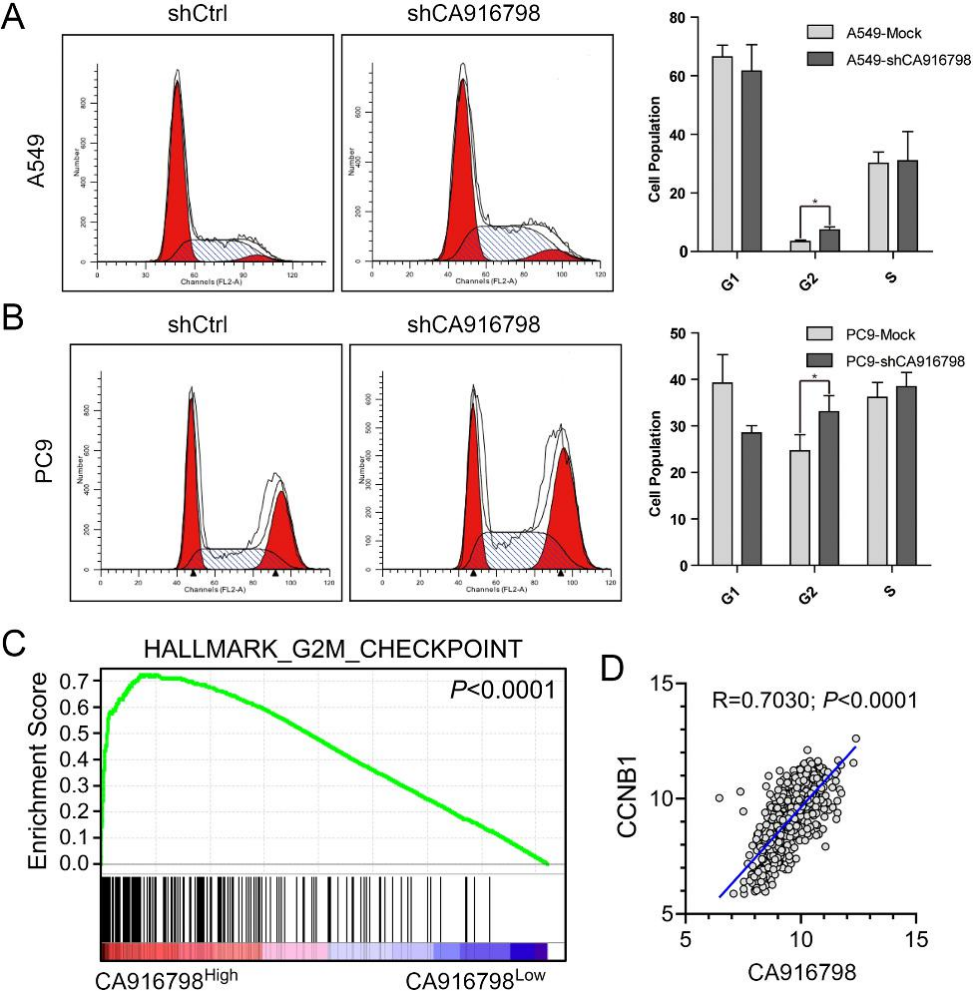
**CA916798 regulates G2/M-phase transition in LUAD cells** **A)** Cell cycle analysis in CA916798-knockdown LUAD cells and mock cells (*P < 0.05) **B)** Cell cycle analysis in CA916798-overexpressing LUAD cells and mock cells (*P < 0.05) **C)** Geneset enrichment assay on TCGA_LUAD dataset **D)** Pearson correlation of CA916798 with CCNB1 from TCGA_LUAD dataset

To further pursue the mechanism on the regulation of LUAD cell growth by CA916798, we examined the effect of CA916798 on cell cycle of LUAD cells through flow cytometric analysis. The result showed that CA916798 knockdown in two LUAD cell lines consistently induced G2/M phase arrest (Fig. 3A and B). GSEA [34, 35] on TCGA_LUAD dataset suggested that high expression of CA916798 significantly enriched HALLMARK_G2M_CHECKPOINT geneset (Fig. 3C). It is known that CCNB1 is the critical checkpoint for G2/M phase [36], and analysis on TCGA_LUAD dataset revealed that CCNB1 was the most correlated gene with CA916798 among all cyclin genes (Fig. 3D **and** Table 4). These findings indicated that CA916798 might regulate the G2/M phase transition in LUAD cells, and silencing CA916798 could suppress tumor growth by inducing G2/M phase arrest.

**Table 4:** Pearson correlation of CA916798 with cyclin genes

Correlation of CA916798	Pearson R	P Value
**vs. CCNB1**	**0.703**	**< 0.0001**
vs. CCNB2	0.6812	< 0.0001
vs. CCNA2	0.6256	< 0.0001
vs. CCNE1	0.5929	< 0.0001
vs. CCNE2	0.5215	< 0.0001
vs. CCNO	0.286	< 0.0001
vs. CCNI2	0.1826	< 0.0001
vs. CCNC	0.1711	< 0.0001
vs. CCNT2	-0.00597	0.8863
vs. CCNH	-0.01291	0.7572
vs. CCNG2	-0.01784	0.6692
vs. CCNA1	-0.1075	0.0098
vs. CCNT1	-0.1118	0.0072
vs. CCNG1	-0.1145	0.006
vs. CCND1	-0.1427	0.0006
vs. CCNI	-0.1722	< 0.0001
vs. CCNB3	-0.1939	< 0.0001
vs. CCND3	-0.2588	< 0.0001
vs. CCND2	-0.3642	< 0.0001

**CA916798 activated the WEE1/CDK1 axis to promote cell cycle progression.**

**Fig. 4:**
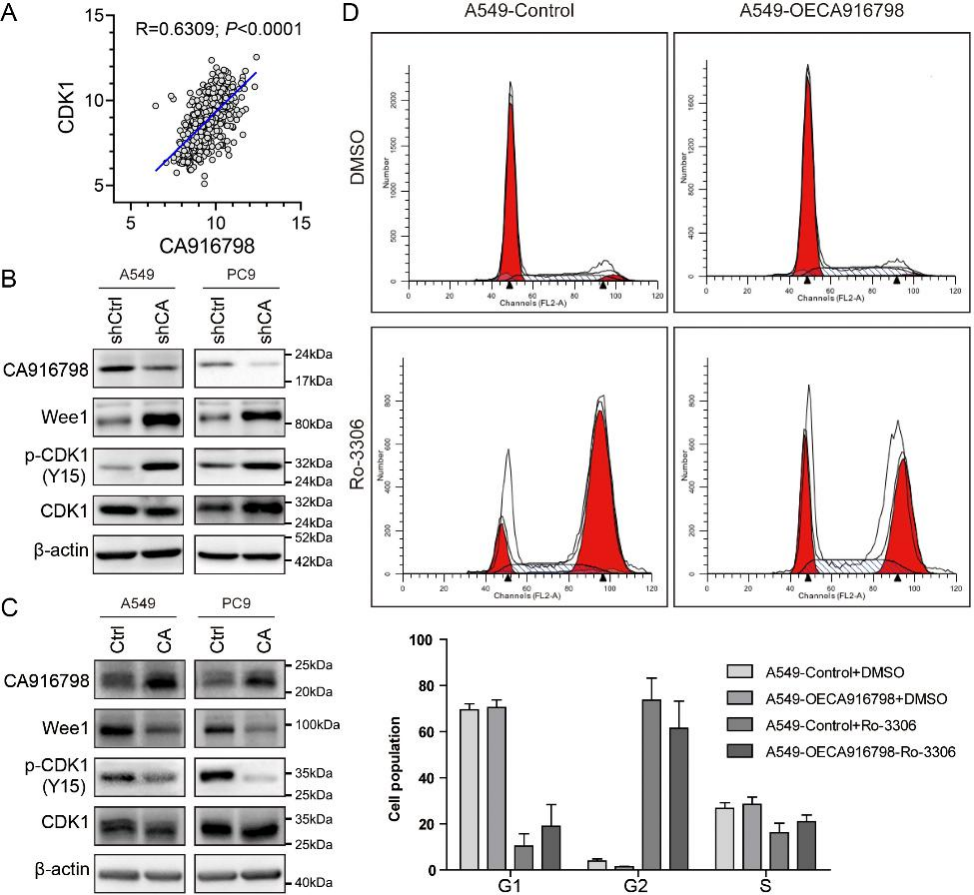
**CA916798 activates WEE1/CDK1 axis to promote cell cycle progression** **A)** Pearson correlation of CA916798 with CDK1 from TCGA_LUAD dataset **B)** Western blotting of A549 and PC9 cells with CA916798 knockdown (shCA) vs. shControl (shCtrl) **C)** Western blotting of A549 and PC9 cells with CA916798 overexpression (CA) vs. empty vector (Ctrl) **D)** Cell cycle distribution measured by flow cytometry in cells treated with 0.1 µM Ro-3306 (CDK1 inhibitor) for 24 h

It is well-known that the CDK1/CCNB1 complex promotes cell cycle progression from G2 phase to M phase and results in cell proliferation [37, 38]. WEE1 inactivates the CDK1/CCNB1 complex by phosphorylating CDK1 at Tyr15 amino acid. Conversely, CDC25C activates the CDK1/CCNB1 complex by dephosphorylate CDK1 at the Tyr15 [37, 38]. Pearson correlation on TCGA_LUAD dataset revealed significantly positive correlation of CA916798 and CDK1 (Fig. 4A). Moreover, protein levels of p-CDK1(Tyr15) and WEE1 were upregulated upon interference of CA916798 in A549 and PC9 cells (Fig. 4B). On the contrary, overexpression of CA916798 reduced protein levels of p-CDK1(Tyr15) and WEE1 (Fig. 4C). These findings suggested that CA916798 might promote proliferation of LUAD cells by inhibiting the expression of WEE1 and the phosphorylation of CDK1(Tyr15). Next, we treated A549 cells with CDK1 inhibitor Ro-33,066 (5 µM for 24 h) and the data indicated that CDK1 inhibitor leaded to G2/M phase arrest in both A549-Control and A549-CA916798 cells (Fig. 4D). Therefore, CA916798 could promote cell proliferation through regulating WEE1/CDK1 axis.

**CA916798 was correlated with Gefitinib sensitivity.**

**Fig. 5:**
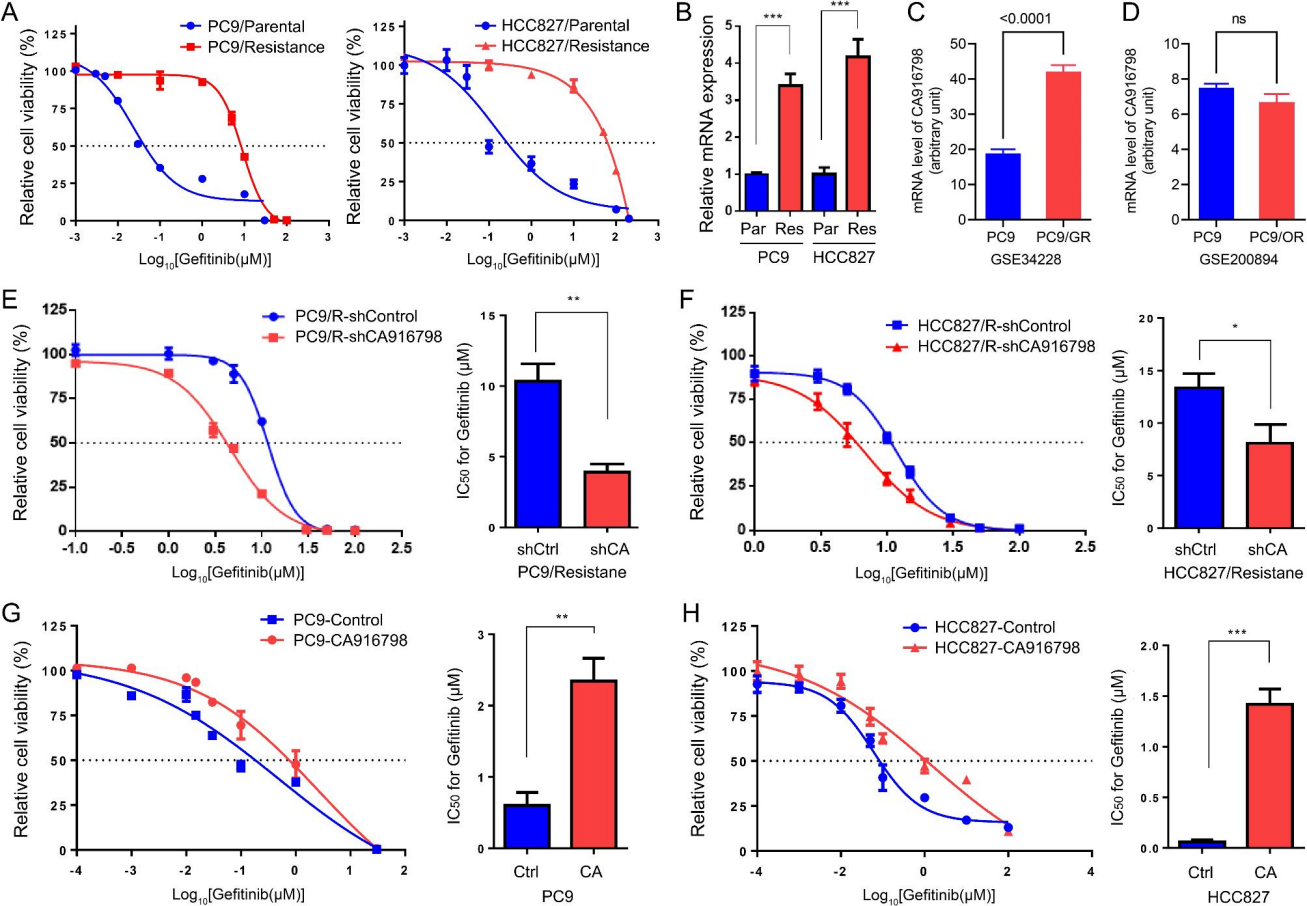
**Correlation of CA916798 with Gefitinib sensitivity in LUAD cells** **A)** Cell growth curves of parent and Gefitinib-resistant cells from PC9 and HCC827 measured via CCK8. **B)** mRNA level of CA916798 in parent and Gefitinib-resistant cells from PC9 and HCC827 measured via qRT-PCR. ***P < 0.001 **C)** mRNA level of CA916798 in PC9 and PC9/GR (Gefitinib resistance) cells **D)** mRNA level of CA916798 in PC9 and PC9/OR (Osimertinib resistance) cells **E and F)** Cell growth curves with Gefitinib treatment and IC_50_ calculation of Gefitinib for PC9/Resistance **(E)** and HCC827/Resistance **(F)** cells stably transfected with control shRNA (shCtrl) or shRNA targeting CA916798 (shCA). **P < 0.01 **G and H)** Cell growth curves with Gefitinib treatment and IC_50_ calculation of Gefitinib for PC9 **(G)** and HCC **(H)** cells stably transfected with empty vector (Ctrl) or CA916798 (CA). **P < 0.01, ***P < 0.001

For LUAD, TKI targeting EGFR, such as Gefitinib, promotes G2/M phase arrest to inhibit tumor growth but fails to do so in TKI resistant cells. Thus, we asked whether CA916798 could facilitate resistance of LUAD cells against Gefitinib. To this aim, we developed Gefitinib-resistant PC9 (PC9/R) and HCC827 (HCC/R) cells through culturing the cells in the presence of Gefitinib with gradually increased concentrations. CCK8 assay showed that IC_50_ of parental PC9 and PC9/R cells for Gefitinib were 0.028 µM and 8 µM, respectively, and IC_50_ of parental HCC827 and HCC/R cells for Gefitinib were 0.141µM and 18.27µM, respectively (Fig. 5A), confirming successful establishment of Gefitinib-resistant cells. Through qRT-PCR, we found that the mRNA level of CA916798 was significantly upregulated in Gefitinib-resistant cells compared with parental cells (Fig. 5B), implying potential implication of CA916798 in acquired Gefitinib resistance. Consistent with our result, analysis on data from GEO dataset GSE34228 also showed that CA916798 expression was increased in PC9/R cells compared with PC9 parental cells (Fig. 5C). In GSE172002, the expression of CA916798 in HCC827/R cells was also higher than that in HCC827 parental cells (FPKM value 56.89 vs. 35.63). However, the analysis on GSE200894 indicated that the sensitivity of PC9 cells to the third-generation TKI, osimertinib, was not associated with CA916798 expression (Fig. 5D). To verify whether CA916798 participated in the regulation of LUAD response to Gefitinib, PC9/R and HCC/R cells were stably infected by lentivirus including shRNA targeting CA916798 (PC9/R-siCA and HCC/R-siCA) or control shRNA (PC9/R-siCtrl and HCC/R-siCtrl) (Fig. 5E F). Notably, knockdown of CA916798 in Gefitinib-resistant cells significantly decreased IC_50_ against Gefitinib from 10.34 µM to 3.92 µM (*P* < 0.01) in PC9/R cells and from 14.1 µM to 8.07 µM (*P* < 0.05) in HCC/R cells, respectively (Fig. 5E F). Furthermore, forced expression of CA916798 in parental PC9 and HCC827 cells increased IC_50_ of the cells against Gefitinib from 0.48 µM to 2.60 µM (*P* < 0.01) in PC9 cells and from 0.06 µM to 1.42 µM (*P* < 0.001) in HCC cells, respectively (Fig. 5G H). Therefore, CA916798 was tightly associated with development of Gefitinib resistance in LUAD cells.

**CA916798 knockdown promoted inhibitory effects of Gefitinib*in vivo***.

**Fig. 6:**
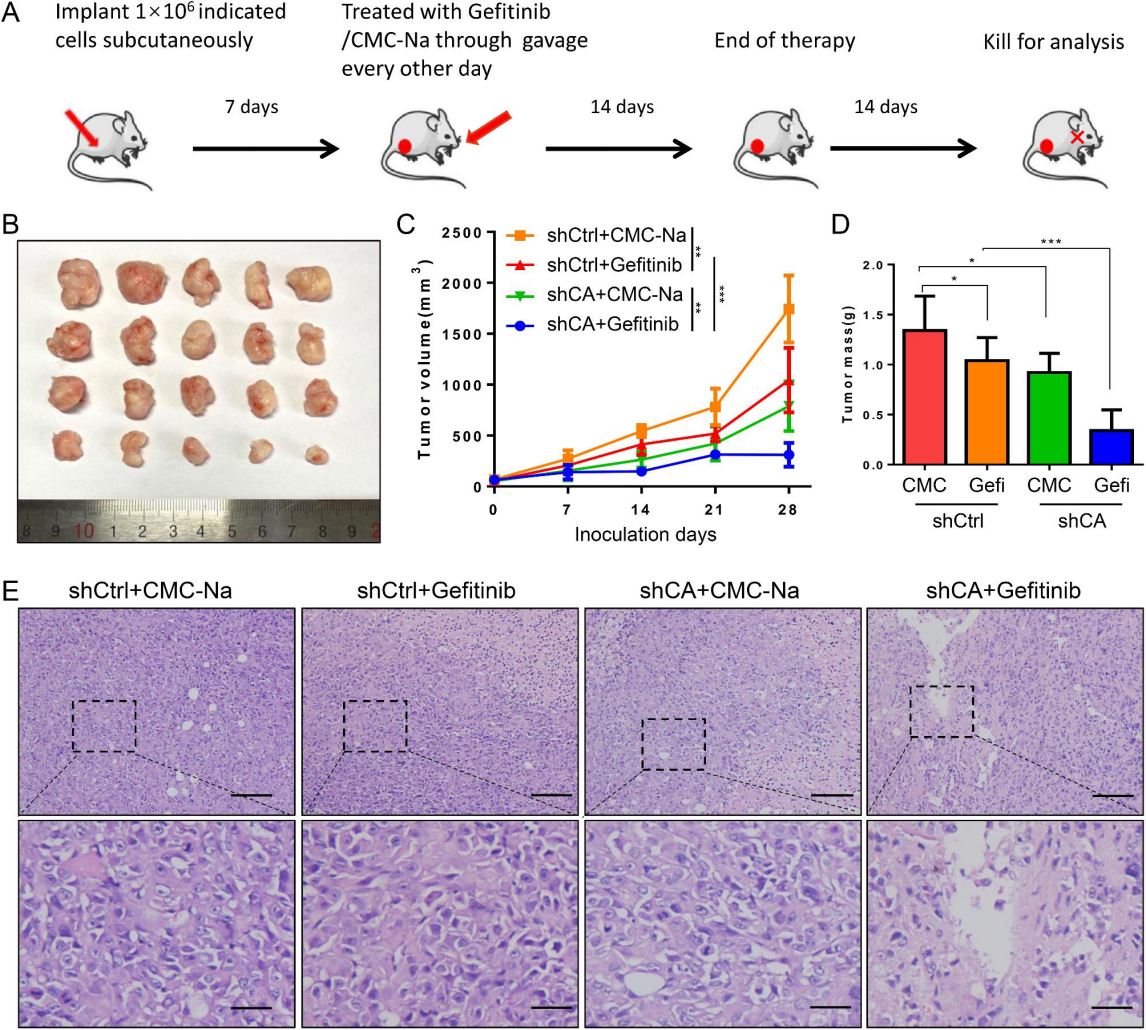
**Targeting CA916798 enhances Gefitinib inhibitory effects on LUAD cells** **A)** Flow chart of in vivo experiment. CMC-Na is used as control for Gefitinib **B-D)** Tumor image (B), tumor volume (C), and tumor weight (D) of xenograft models generated using PC9/GR cells stably transfected with control shRNA (Mock) or shCA916798 treated with CMC-Na or Gefitinib. Data are shown as mean ± SD (n = 5, *P < 0.05, ***P < 0.001 **E)** Representative HE staining of xenograft tumors. Scale bar = 100 μm (upper panels) or 25 μm (lower panels)

Since overexpression of CA916798 dampened effects of Gefitinib on LUAD cells, we then examined whether interference of CA916798 expression would enhance Gefitinib inhibitory effects. For this purpose, we established a xenograft model through subcutaneously inoculated PC9/R-shCA916798 or PC9/R-shCtrl cells into NOD/SCID mice followed by treatment of Gefitinib or placebo (Fig. 6A). Consistent with in vitro results, CA916798 knockdown significantly inhibited the growth of xenograft (Fig. 6B). Both tumor volume and tumor weight were decreased with knockdown of CA916798 (Fig. 6C and D). Moreover, Gefitinib showed stronger inhibitory effects on PC9/shCA916798 cells than PC9/shCtrl cells (Fig. 6B and D). HE staining indicated that in Gefitinib treatment did not obviously change pathological features of PC9/shCtrl cells and only slightly decreased density tumor cells (Fig. 6E). However, Gefitinib treatment induced significant death of tumor cells and collapse of nuclei (Fig. 6E), confirmed the inhibitory effects of Gefitinib on LUAD cells with knock down of CA916798. Therefore, diminishing CA916798 might augment inhibitory effects of Gefitinib on LUAD cells.

## Discussion

**Fig. 7:**
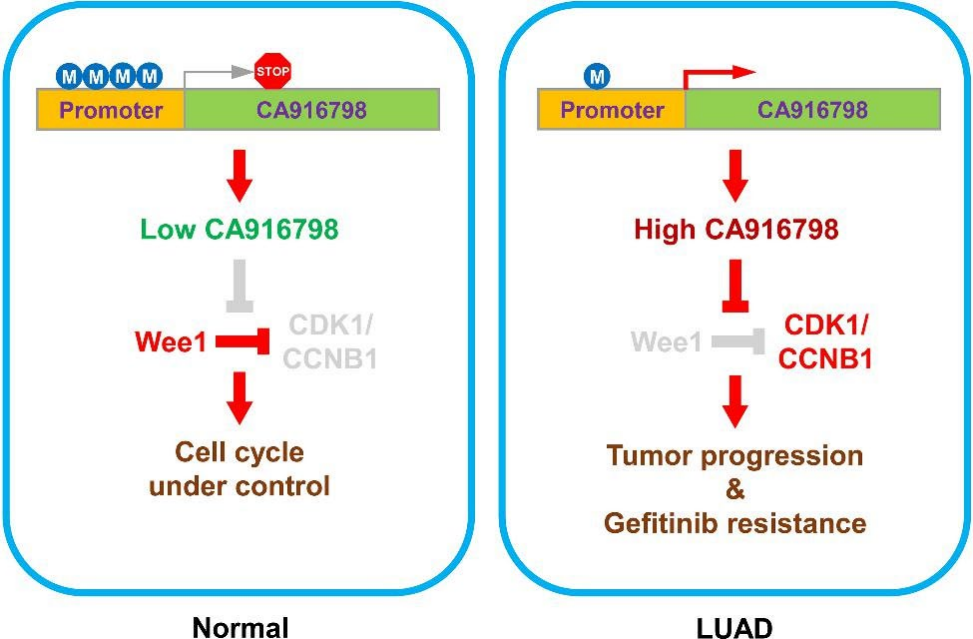
**Targeting CA916798 enhances Gefitinib inhibitory effects on LUAD cells** Schematic diagram of CA916798-mediated progression and Gefitinib resistance of LUAD. In normal lung epithelial cells, promoter of CA916798 gene is hypermethylated and the transcription level of CA916798 is low, which leads to suppression of CDK1 by WEE1 and cell cycle arrest (left panel). In LUAD cells, promoter of CA916798 gene is hypomethylated and the transcription level of CA916798 is dramatically elevated. In this context, CA916798 blocks the inhibition of CDK1 by WEE1 and facilitates cell cycle progression, resulting in LUAD progression and Gefitinib resistance (right panel)

In this study, we explored the clinicopathological significance of CA916798 in LUAD and found that CA916798 expression was significantly correlated with poor prognosis of LUAD patients. Surprisingly, our study also identified a key methylation site in promote region of CA916798 gene as a mechanic regulator for CA916798 gene expression and clinical predictor for patient prognosis of LUAD. Through cell and animal experiments, we further delineated that CA916798 promoted proliferation and tumorigenesis of A549 cells. In addition, CA916798 was upregulated in Gefitinib-resistant LUAD cells and elimination of CA916798 facilitated Gefitinib effects on LUAD cells (Fig. 7).

The occurrence and development of NSCLC is a complex process involving multiple abnormal gene expression and mutation. EGFR-tyrosine kinase inhibitors have opened up a new era of treatment in NSCLC [39, 40]. Subsequent drugs targeting ALK, ROS1, KRAS, BRAF, MET, RET, NTRK, and other genes have also been explored [41]. As more genes related to lung cancer are discovered, more targeted drugs will enter clinical application, expanding the treatment possibilities for lung cancer patients.

The CA916798 gene was identified more than 10 years ago, but the function of its encoded protein is still obscure. It is found that the CA916798 gene encodes a minor histocompatibility antigen, which is presented to cytotoxic T cells and initiates killing effects on tumor cells in patients with renal cancer [21]. The CA916798-encoded antigen is expressed in a variety of tumor cells, so it may be related to tumor immunity. However, this possibility remains to be investigated. We previously reported that the CA916798 gene is related to chemotherapy resistance in lung cancer; a higher the expression of this gene was associated with a higher chemotherapy resistance. Further studies showed that the gene is regulated by the PI3K/AKT signaling axis, which regulates the proliferation and apoptosis of malignant tumors. Therefore, CA916798 gene might be a lung cancer-related gene and represent a promising therapeutic target for LUAD treatment.

Cell proliferation is tightly regulated by a cell cycle network, including cyclin proteins, cyclin-dependent protein kinases, and cyclin-dependent protein kinase inhibitors. G1/S and G2/M boundaries are the most important checkpoints in cell cycle regulation. Our results showed that CA916798 influenced the expression of WEE1 and CDK1, which are key molecules that regulate the G2/M transition. It is clear that CDK1 binds to CCNB1 as a mitotic promoter that drives G2 to M phase transition. The mitotic promoter complex is inactive due to phosphorylation of Thr14 and Tyr15 of CDK1 by WEE1. We found that CA916798 inhibited the expression of WEE1 in LUAD cells, suggesting that CA916798 might be a transcriptional repressor of WEE1. Reduction of WEE1 promotes dephosphorylation and activation of CDK1 and eventually leads to cell cycle progression. These findings indicated that CA916798 promoted cell proliferation through regulation of WEE1/CDK1.

EGFR-TKI inhibits cell proliferation by competitively binding to ATP binding sites in the catalytic domain of tyrosine kinase, inhibiting its self-phosphorylation and blocking downstream signal transduction. In vivo, Gefitinib and other EGFR-TKI drugs widely inhibit the tumor growth of human tumor cell derived lines xenografted in nude mice, and improve the anti-tumor activity of chemotherapy, radiotherapy and hormone therapy. Our data firstly reported that knockdown of CA916798 improved the sensitivity of drug-resistant cells to EGFR-TKI and enhanced the growth inhibition of EGFR-TKI on NSCLC cells. LUAD cell derived xenograft in immunodeficient NOD/SCID mice further established that knockdown of CA916798 combined with TKI significantly inhibited tumor growth compared with single drug treatment. These findings suggested that the increased expression of CA916798 significantly promoted the development of EGFR TKIs resistance, so CA916798 might be an important molecule to promote the acquired EGFR TKIs resistance of LUAD cells. However, it should be mentioned that Gefitinib is a first-generation TKI, while Osimertinib is a third-generation TKI. Our current finding, in association with other reports, emphasized that CA916798 is associated with Gefitinib but not Osimertinib resistance. Since first-generation TKIs block EGFR activity through reversible interaction, CA916798 might lead to Gefitinib resistance through interference with the interaction between EGFR and Gefitinib. However, third-generation TKIs specifically target mutant EGFR, which could not be affected by CA916798 function. Thus, it would be worthy further pursuing the differences of CA916798 function and regulation in LUAD cells with wild-type EGFR *versus* mutant EGFR.

Together, our work herein demonstrated that CA916798 promoted LUAD cell proliferation and was related with poor prognosis of LUAD patients probably through inhibiting WEE1 expression and subsequent activation of CDK1. Thus, CA916798 could be considered as a promising prognostic marker and a therapeutic target for LUAD.

## Electronic supplementary material

Below is the link to the electronic supplementary material.

Supplementary Material 1

## Data Availability

All data and materials are available in the manuscript. TCGA_LUAD dataset and TCGA_LUAD methylation 450 K datasets are downloaded from https://xenabrowser.net/. Kaplan-Meier plotter of Lung Cancer are available at https://kmplot.com/analysis/.

## List of abbreviations

LUAD: lung adenocarcinoma
NSCLC: non-small cell lung cancer
TKIs: tyrosine kinase inhibitors
shRNAs: short hairpin RNAs
IHC: immunohistochemistry
IOD: integrated optical density
PBS: phosphate buffer saline
GSEA: Gene Set Enrichment Assay
HE: Hematoxylin-eosin.
