# Supplementary figures and images for: CA916798 predicts poor prognosis and promotes Gefitinib resistance for lung adenocarcinoma
Source: BMC Cancer. 2023 Mar 23;23:266. doi: 10.1186/s12885-023-10735-3 (PMC10035219; doi:10.1186/s12885-023-10735-3)

**Fig. 4B – CA916798**

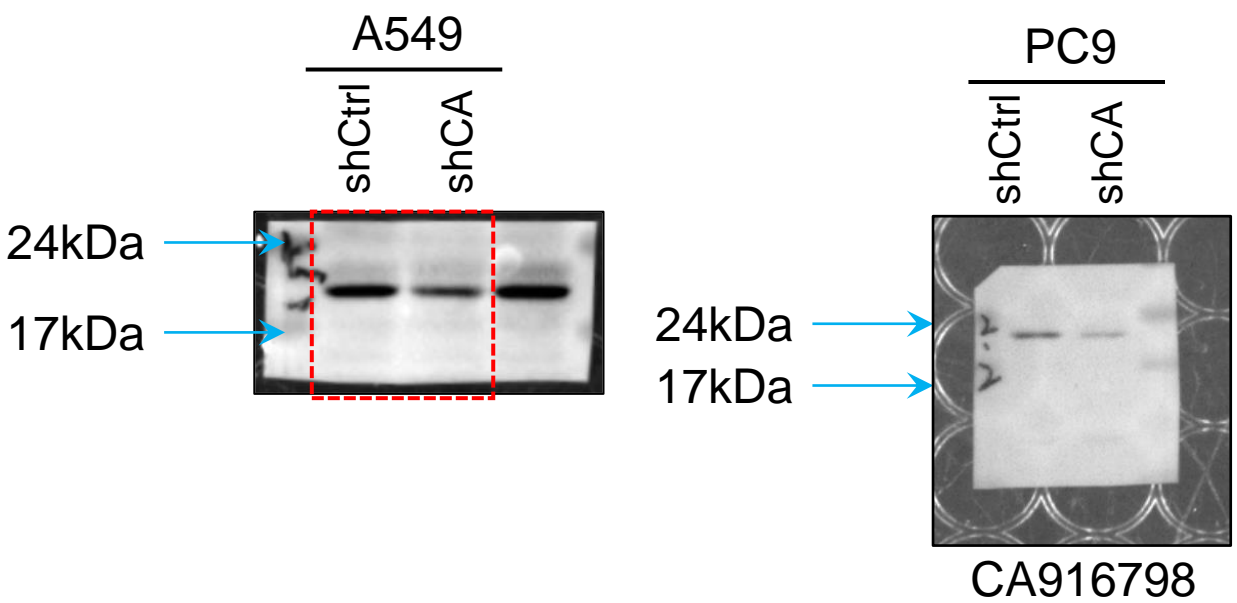

**Fig. 4B – Wee1**

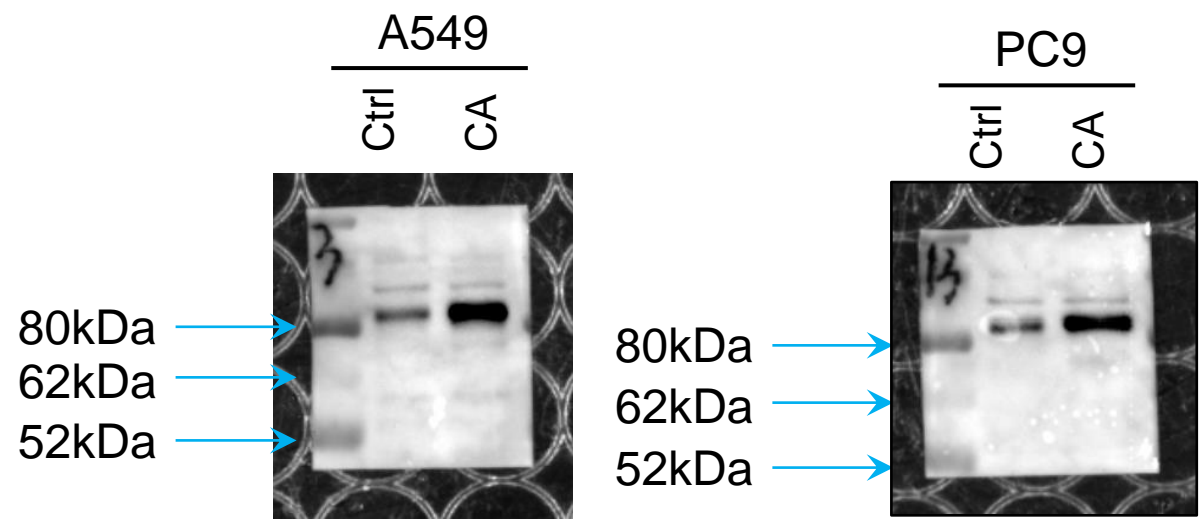

**Fig. 4B – p-CDK1(Y15)**

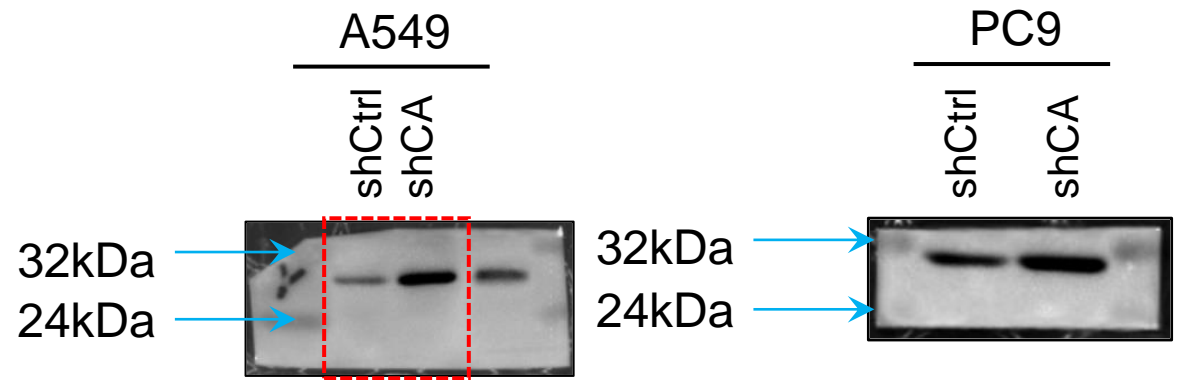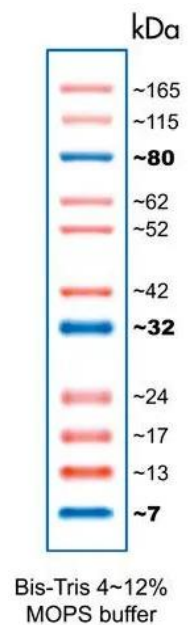

**Fig. 4B – CDK1**

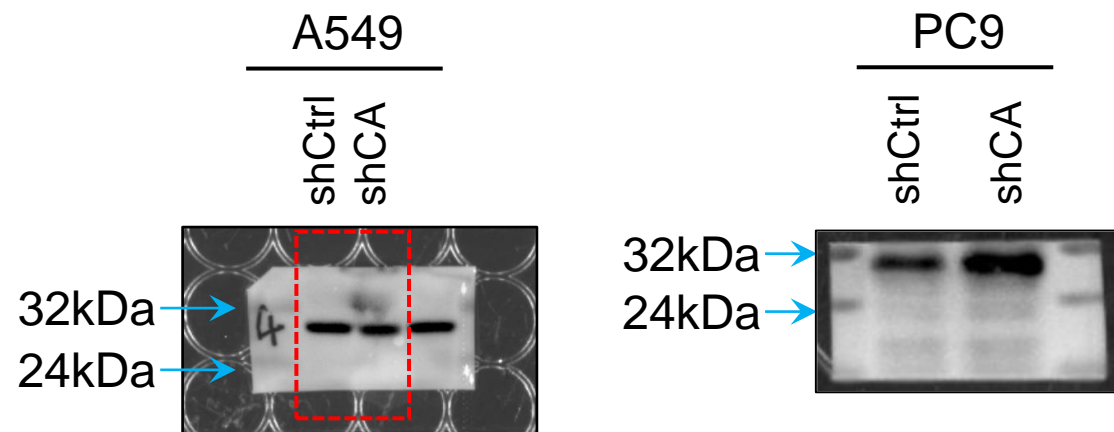

**Fig. 4B – actin**

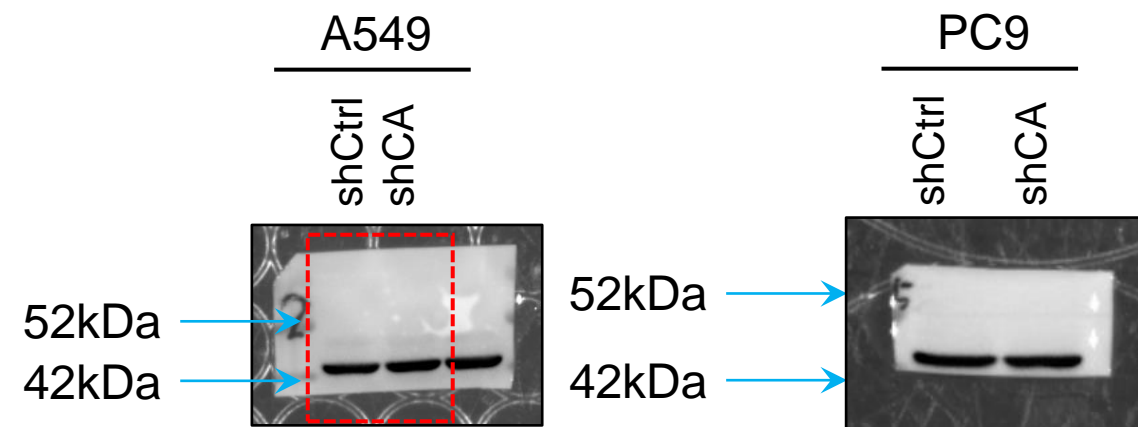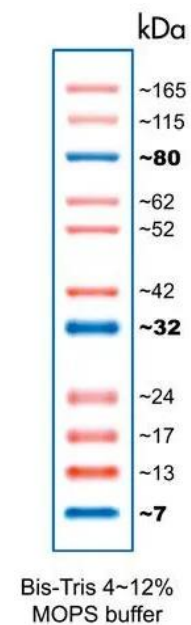

**Fig. 4C – CA916798**

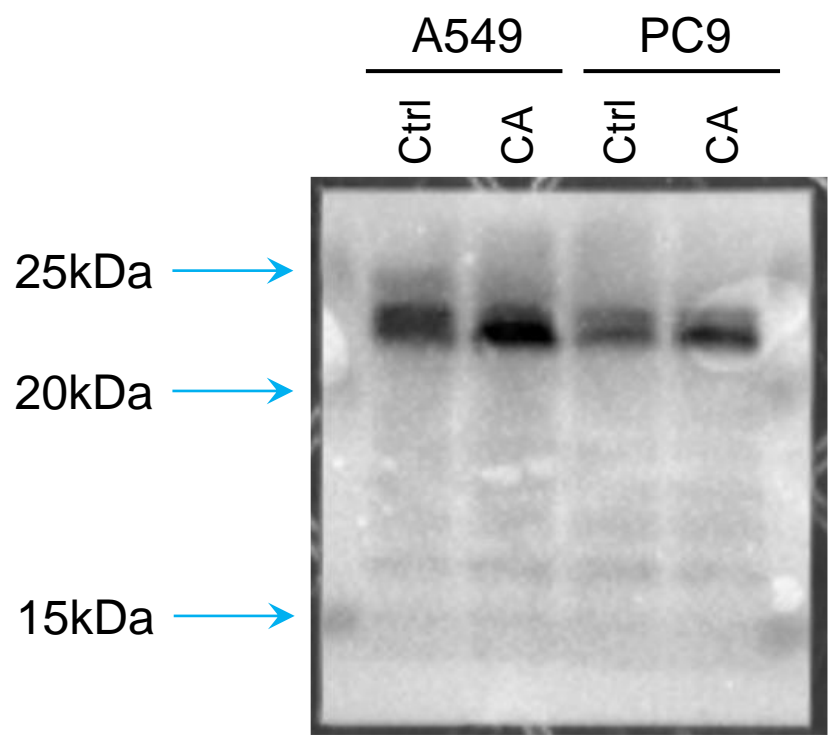

**Fig. 4C – Wee1**

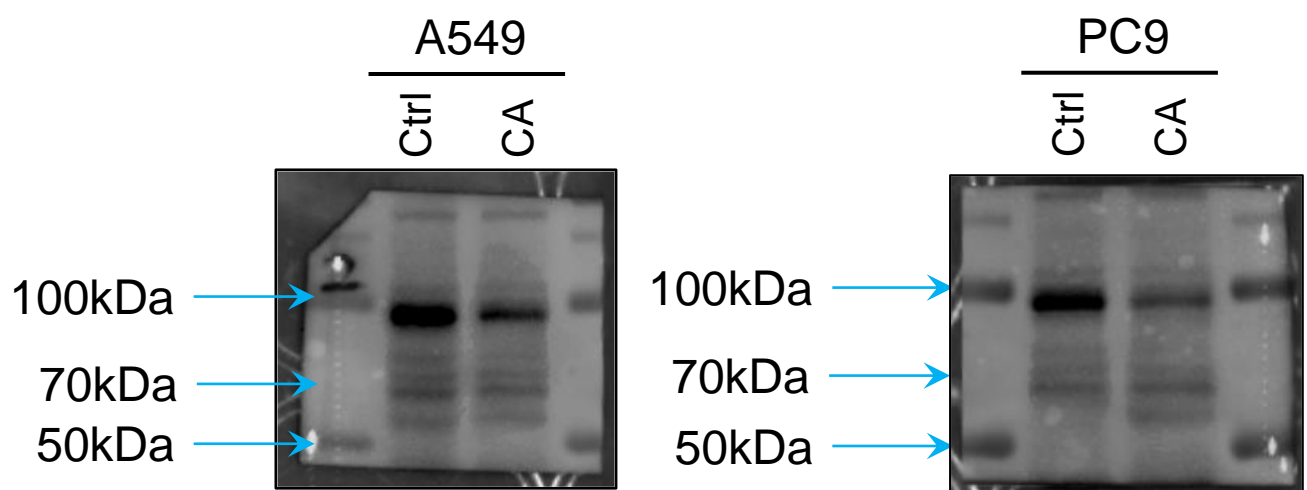

**Fig. 4C – p-CDK1(Y15)**

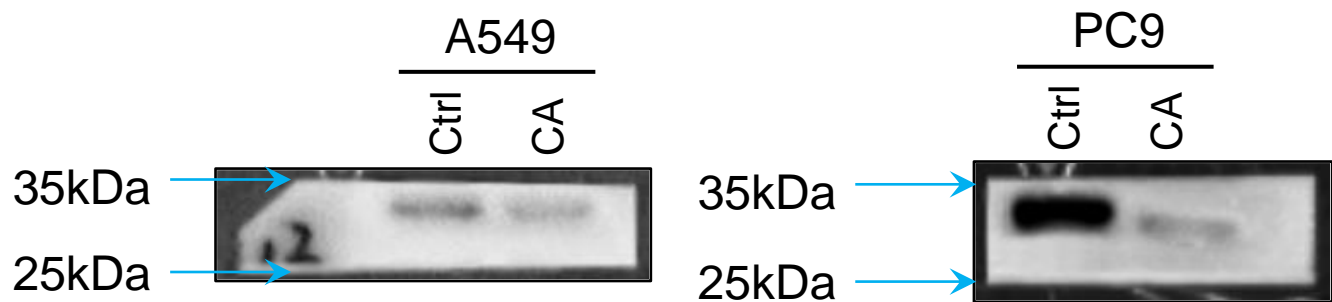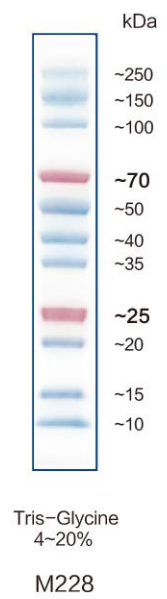

**Fig. 4C – CDK1**

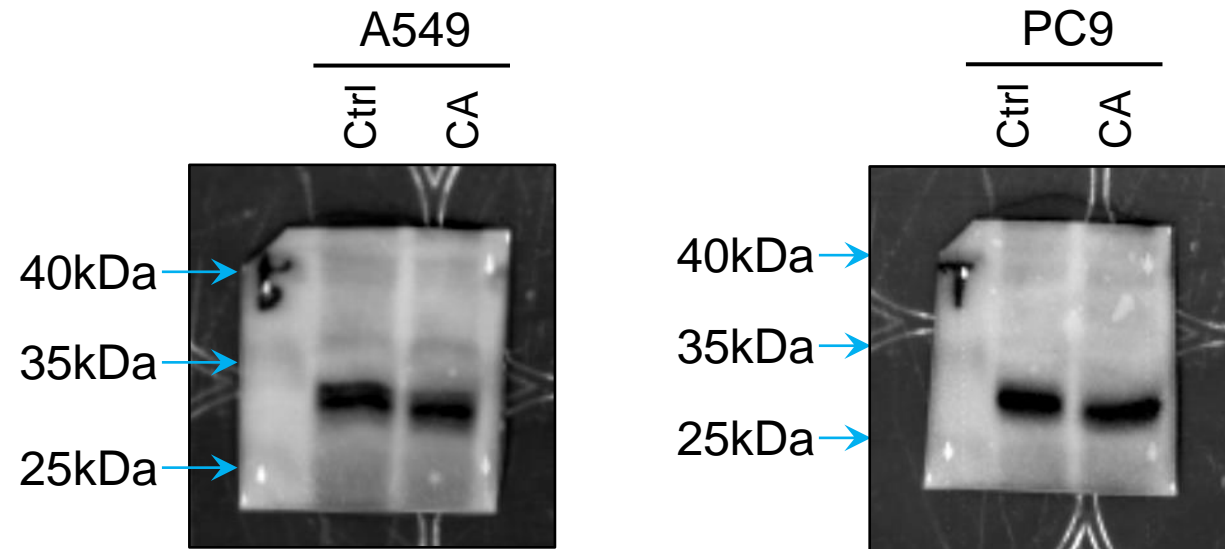

**Fig. 4C – actin**

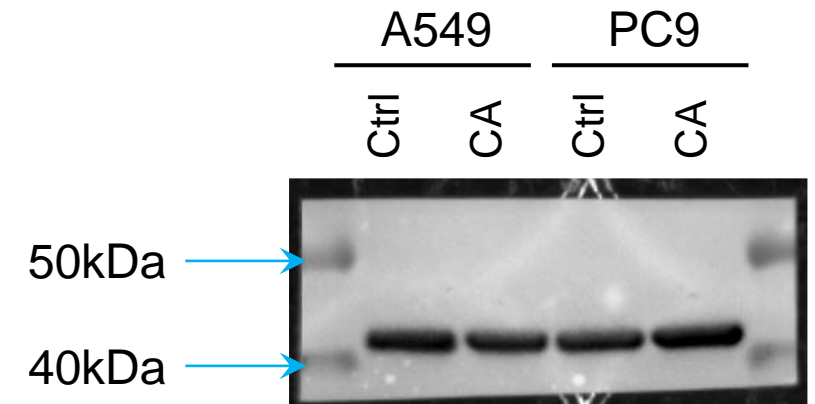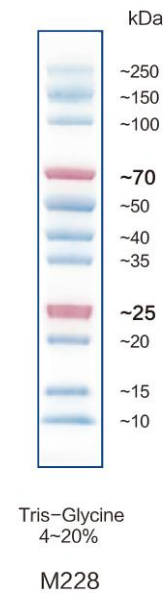

Supplement: Supplementary file 1 — Supplementary Material 1 [file 12885_2023_10735_MOESM1_ESM.pdf]
